# Recent Advances in Smellscape Research for the Built Environment

**DOI:** 10.3389/fpsyg.2021.700514

**Published:** 2021-07-19

**Authors:** Jieling Xiao, Francesco Aletta, Antonella Radicchi, Kate McLean, Larry E. Shiner, Caro Verbeek

**Affiliations:** ^1^School of Architecture and Design, Birmingham City University, Birmingham, United Kingdom; ^2^Institute for Environmental Design and Engineering, The Bartlett, University College London, London, United Kingdom; ^3^Institute of Urban and Regional Planning, Technical University of Berlin, Berlin, Germany; ^4^School of Creative Arts and Industries, Canterbury Christ Church University, Canterbury, United Kingdom; ^5^Faculty of Visual Arts, University of Illinois Springfield, Springfield, IL, United States; ^6^Department of History, Medical and Health Humanities, Vrije Universiteit Amsterdam, Amsterdam, Netherlands

**Keywords:** smellscape, odour policy making, olfaction, built environment, art and design, museum, citizen science, technology

## Abstract

The interrelationships between humans, smells and the built environment have been the focus of increasing numbers of research studies in the past ten years. This paper reviews these trends and identifies the challenges in smellscape research from three aspects: methodological approaches, artistic design interventions and museum practices, and odour policy making. In response to the gaps and challenges identified, three areas of future research have also been identified for this field: smell archives and databases, social justice within odour control and management, and research into advanced building materials.

## Introduction

This paper describes recent advances in smellscape research and offers recommendations for future work to scholars, practitioners and artists. The smellscape concept was introduced by Porteous (1985), who suggested that perceptions of smell, while comparable to spatially ordered visual impressions, differ in their episodic nature, and academic interest in the concept and its analysis and design approaches has grown over the past 30 years. Henshaw (2014, p. 5) supplemented Porteous's definition of smellscape, describing it as “referring to the overall smell environment, but with the acknowledgment that as human beings, we are only capable of detecting this partially at any one point of time, although we may carry a mental image or memory of the smellscape in its totality.” The word *smell* in this concept differs from *odour—*the combined substances in the air that cause olfactory sensations—as it emphasizes the human experience as a perceptual construct (Xiao et al., 2018a).

**Figure 1:**
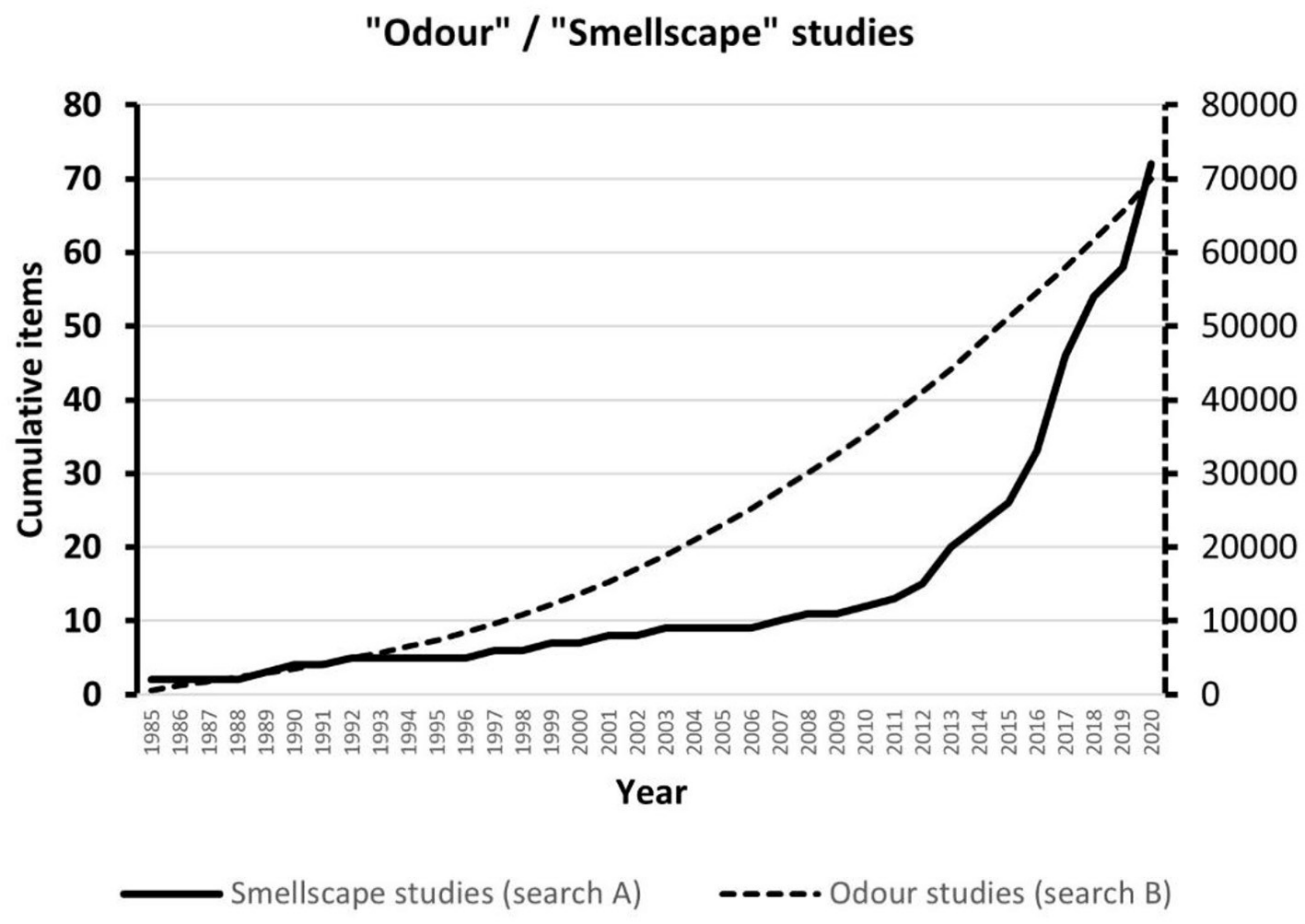
Cumulative number of scientific publications on smellscape and the built environment (search A) and odour research in general (search B). Query string in Scopus for search A: TITLE-ABS-KEY (smellscap*) OR TITLE-ABS-KEY (smell) AND TITLE-ABS-KEY (“built environment”). Query string in Scopus for search B: TITLE-ABS-KEY(odour).

A search of the Scopus database (Figure 1; the search terms are reported in the caption) aimed to find scientific publications at the intersection of smellscape and built environment studies. To provide a baseline, a second search used only “odour” as a keyword. The numbers of the two queries differed by three orders of magnitude, but, while the latter shows a relatively stable linear growth over time, the former has grown exponentially in the past 10 years, which likely reflects a growing interest in the concept of smellscape as a user-centred approach in the built environment community. Three main themes emerged from a qualitative review of the titles and abstracts of the smellscape-related search:

- smellscape as intangible heritage and an element of historical and cultural value: studies dealing with scents of the past, smell geographies, historical reconstructions and smells in museums and exhibitions (~20 papers)

- smellscape as a therapeutic or experiential element: studies related to laboratory experiments for environmental assessment, sensory-deprived people and smell as an element for tourism and well-being (~22 papers)
- smellscape as a design approach for the built environment: studies dealing with design and perception models, smell mapping and representation and smell as a place-making element and component of the public space (~28 papers)

All these themes raise similar methodological challenges in dealing with what is possibly the least tangible dimension of human life in the built environment. Similarly, they all highlight the interest in not leaving the smell experience to chance but rather considering it as a designable element at both the small (i.e., space, building) and large (i.e., city) scale.

## Methodological Approaches in Smellscape Research

In urban and architectural studies, smellscape research takes qualitative approaches to consider the social and psychological impacts of diverse smells from peoples' *in situ* experiences or recalled memories. Methods such as smellwalks, interviews, observations and the scale rating of perceptual factors (i.e., like/dislike, familiar-unfamiliar) are commonly employed to collect data on people's experiences and subjective evaluations of the olfactory environment in real contexts. Recent years have seen growth in laboratory experiments using virtual reality technologies to test design hypotheses or perceptions of existing spaces through multisensory interactions with two or more modalities involving the sense of smell (see Jiang et al., 2016; Ba and Kang, 2019a). Although such experiments have revealed the impossibility to fully replicating a smell environment, these methods are useful for testing the impact of adding a specific smell to an existing context. For example, Doukakis et al. (2019) proposed an audio-olfactory-visual environmental setting that enables a controlled olfactory impulse at various concentrations through computational programming and simulation. In addition to the use of subjective data, objective data from electroencephalography, eye-tracking, heart rate variations and the galvanic skin responses used in neuro-biological studies are also emerging in laboratory experiments to study people's stress levels and emotional responses (see Spence, 2020a; Jiang et al., 2021; McEwan et al., 2021).

The biggest challenge to the advance of smellscape research and practice in the built environment is the lack of applicable smell databases to aid the design process and predict outcomes. Spatial-odour relationships can be simulated through air flow simulation software, such as CFD, AERMOD and CULPUFF, but sampling odour on site to enable such simulations is still not a straightforward process, as it requires laboratory analysis by chemistry specialists (see EN 13725:2003). In urban planning practice, simulations of odour dispersion and concentration are mostly limited to the sites of industrial or waste management plants, and they target specific odorants. By contrast, the smellscape approach considers the implications of smells in spatial design to create a positive impact on human well-being by both controlling negative odorants and introducing scents into spaces. A data set per unit of the odorants in everyday sources of smell—objects, plants and foods as well as cosmetic and cleaning products—would be needed to aid in the prediction of the total smell environment to support spatial planning and design. Harel et al. (2003) proposed a three-component system called iSmell for creating a smell database, including a sniffer space (the odorants in the ambient environment), a sensory space (the detection of odorants) and a psychophysical space, but no further development of the project has been reported. Obrist et al. (2014) summarized 10 categories of people's associations of smells in everyday life by analyzing participants' smell stories. These can be used to develop future human-computer interaction systems that match spaces, smells and situations with potential olfactory perceptions as a reference for designers to create more pleasant smell experiences, enhance storytelling and avoid embarrassing moments caused by smells. For example, in the positive smell stories, the most frequent words used to describe the quality of smells perceived are pleasant (60%), clean (42%), sweet (38%) and fresh (31%).The associated context and personal data can be traced via the database. A designer intending to design a space that smelt fresh and clean could refer to the spatial form, social conditions and actual smell experiences from those stories to determine its design elements. However, a smell data set that matches smells, experiences and behaviors would be essential to achieving that goal.

The practice of smell mapping takes an anthropocentric approach to the detection, description and depiction of temporal- and location-specific olfactory environments (McLean, 2019). Situated within communication design, smell mapping deploys the smellwalk as a method of data collection and to create subsequent representational formats to illustrate subjective, dynamic, vernacular urban smellscapes (Henshaw, 2015). Normally conducted with up to 20 people who sniff passing air and close-up objects, the smellwalk invites participants to foreground their olfactory sense and temporarily attend to an alternative sensory modality, but the global Covid-19 pandemic presented immediate challenges to the practice of large-group smellwalking. While Covid-19 precautions vary, a responsible approach would limit group sizes in accordance with government guidance and restrictions. The reduced group sizes, however, would result in the collection of less fruitful data on people's *in situ* experiences of the temporary time-space settings specific to the walk, and the urban smellwalk methodology includes active sniffing in both indoor and outdoor spaces, which might necessitate wearing face masks. The choice of material and fitting of face masks would likely alter the detection of subtle odours.

**Figure 2:**
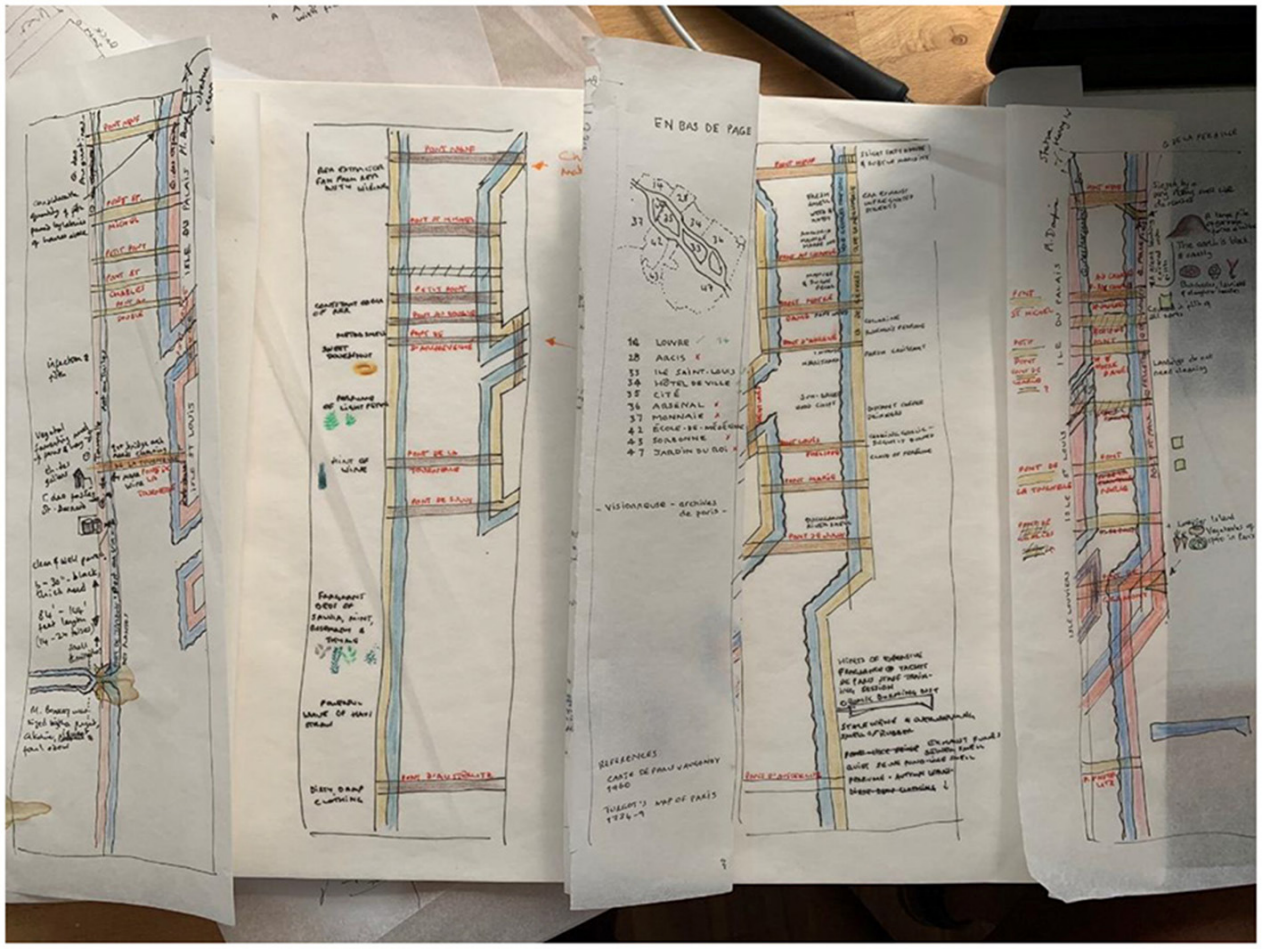
Deploying fictional itinerary mapping practice techniques to depict a historical smellscape alongside a contemporary version (© Kate McLean, 2020).

Future research agendas for smell mapping and olfactory representation may comprise four major strands: cross-modal, historical, digital and future archive. Cross-modal approaches are crucial to understanding human interaction with the smellscape (Ndichu, 2020; Spence, 2020b). Historical smellscapes combine olfactory descriptors, local context and human habituation (see Kiechle, 2017; Dugan, 2018; Tullett, 2019), and both cross-modal and historical smellscapes benefit from multilayered mapping design approaches (Figure 2) that are sympathetic to the era and narrative being depicted (McLean, 2020a,b). Any future archive of smell will need to identify the criteria for olfactory selection and preservation (see Bembibre and Strlič, 2017) and determine the mechanisms for representation. While the Odeuropa (2020) project uses Artificial Intelligence (AI) to collect a database of smell references, the use of digital technology in smell representation requires further work to explore how digital platforms might work to empathically record and convey human experience.

## Rediscovering Designed Smellscapes

Many public officials think of odours only as something to be controlled or eliminated, and even many urban designers overlook smells' positive contributions to health and aesthetic satisfaction (Henshaw, 2014). This neglect of the benefits of the urban smellscape no doubt partly results from the traditional disparagement of the sense of smell in Western culture, but it is also an extension of modern sanitary campaigns that led to the relative deodorisation of most Western cities (Classen et al., 1994, p. 78; Jütte, 2005, p. 267). Unfortunately, some people mistake any odour they find personally distasteful for pollution. But one needs to distinguish pollutants—chemicals in the air that can cause actual harm, but that may or may not be detectable by their odour, such as carbon monoxide—from odourants—molecules that are by definition detectable but may cause no harm at all. Conversely, chemicals such as carbon dioxide- in normal conditions undetectable by its odour but may cause physiological consequences- are often ignored. Among the unfortunate effects of a focus on deodorisation and control is the increasing olfactory blandness of some upscale areas of many cities, which often contributes to a loss of the sense of place (Meighan, 2008).

If urban designers were to become more alert to the positive contributions of distinctive smells, they might not only begin to regard many existing odours as assets rather than nuisances but also begin to create new smellscapes that enhance a sense of well-being and aesthetic satisfaction (Pálsdóttir et al., 2021). Henshaw (2014, p. 5) speaks of these benefits as part of the “restorative” use of odours, a concept familiar from environmental psychology studies showing the benefits of exposure to nature. Such studies suggest the generous use of fragrant trees, green spaces, parks, waterways, ponds and fountains in urban design (Xiao et al., 2018b). Ba and Kang (2019b) also suggest that the olfactory experience of a fragrant tree can reduce the annoyance of traffic noise pollution. In the past, such features have been treated primarily as visual objects. Even then, they were often resisted by local governments because of their maintenance expense.

More controversial is the possibility of scenting public spaces with artificially created odours, which, considering the variability of odour preferences and sensitivities, may not be a good idea. Moreover, because a pleasant odour raised to a high intensity can become unpleasant, spreading a fragrance of sufficient concentration to mask traffic or other smells might make the fragrance as offensive as the exhaust fumes. Whatever interventions designers make, they will have to consider not only the appropriate intensity levels but also what types of smells will be consistent with the locale and acceptable to most residents. The practice of smell management and design should also consider individuals' personal and cultural resonances through behaviours and bodily movements in spaces (Wareen and Riach, 2018).

Artists also have a role to play in showing residents both the realities and possibilities of urban smellscapes. Among the most notable interventions of this kind have been the smellwalks conducted by Sissel Tolaas and Kate McLean, who have subsequently used the results to make artworks that are displayed in galleries and museums (Shiner, 2020, p. 278–281). The Australian artist Cat Jones produced an art installation for the 2017 Sydney Festival by interviewing 10 prominent cultural leaders about the city's past and present smellscapes and then playing the interviews for the public, accompanied by her imaginative recreation of the mentioned smells (Leimbach, 2017).

Considering possible applications of the smellscape approach in indoor contexts, a growing number of museums have become aware of a mismatch between their collections and their ocular-centric presentation to audiences. Consequently, visually oriented art museums, such as Amsterdam's Rijksmuseum, the Louvre in Paris and New York's Metropolitan Museum of Art, have integrated historically informed scents in their tours and displays. As challenging and contradictory as it may seem to address the nose in museums of visual art (Clapot, 2019), this approach has many advantages. Olfactory storytelling can add tremendously to the appreciation of the historical context of displayed objects and enhance the feeling of being momentarily transported to another time and place (Stevenson, 2014). Olfactory stimulation in museum contexts also influences behavior, with people becoming much more talkative during and after museum tours, indicating a heightened level of engagement (Spence, 2020c; Verbeek, 2020). Furthermore, blind and partially-sighted people indicate that sounds, tactile impressions and smells help them (re)imagine historical events and point to the utility of (smell-related) objects, which can enhance a sense of inclusivity, accessibility and therefore well-being.

## Odour Policy-Making and the Urban Environment

The policies for odour control and management across Europe present several patterns. They predominantly address odour pollution caused by waste, traffic, plants, soil and water contamination, landfills, livestock facilities and cooking fumes in food districts that can negatively affect quality of life, causing complaints about smell nuisances similar to noise pollution (Bull, 2018). The policies are disparate and even completely absent in many places and lack common criteria for establishing odour-impact thresholds (Rfenacht et al., 2019). The focus remains on measuring emissions at the source, and methods such as evaluating odour impact on receptors (citizens) (e.g., British Standards, 2016) are time consuming, often expensive and still do not provide real-time information on the discomfort of the impacted citizens (Rfenacht et al., 2019). Overall, a top-down approach is usually implemented that fails to actively involve stakeholders in odour management processes, which Henshaw (2014) suggests should be the first step taken.

Against this backdrop, recent advances in smell research can be identified that address these limitations. Expanding on Henshaw (2014), Xiao (2018) proposes an integrated smellscape design process framework grounded on the understanding of smellscape as a perceptual construct. This framework indicates how the five domains in the existing urban design and planning practice can be integrated to implement a participatory process for the analysis, assessment and planning of smellscapes. Another innovative approach to promoting public participation in odour policy-making is to use citizen science tools. For example, the D-NOSES projects collect data on odour pollution through a free mobile app called OdourCollect that enables citizen participation in odour mapping to foster a bottom-up, multi-level governance model in local decision-making.

Finally, existing policies lack a distinction between pleasant and unpleasant odours, and odour is mainly considered a negative element of the urban environment (Bull, 2018). This is reflected in odour control and management strategies that usually apply masking and deodorisation techniques (e.g., Henshaw, 2014; Bull et al., 2018). Conversely, the positive impacts of smells should also be acknowledged in current policy and urban practice. Smells in fact bring distinct identities to places, connect people emotionally to their surroundings, positively influence human behavior and emotion and evoke memories of the past.

## Outlook and Concluding Remarks

Smells, either negative or positive, should be considered as an essential aspect of the design and planning framework of public spaces alongside other sensory components, such as sound and lighting, to create a pleasant, healthy environment (Xiao, 2018). Moving from odour nuisance control to smellscapes opens up opportunities to appreciate the benefits of smells in human experiences and well-being. The gaps revealed through our review of the existing literature include a lack of databases and archives of smells; a lack of place-related histories and meanings; a lack of applicable design frameworks, building materials, scenting systems and representation methods; and limitations in how current policy-making considers and manages smells. Given these gaps and limitations, three directions are suggested for future research on smells and the build environment.

Firstly, setting up smell archives and databases may demand a large, collaborative effort and considerable time in the coming years. Records of smells and odorants provide essential information for replicating and creating a smell environment. The cultural and social value of smell is an important part of the history of towns and cities (Reinarz, 2014), so smell maps and oral stories should be collected to gather the information. Benchmarks of odour concentration and composition and measures of smells' emotional impacts on human experience can be tested in controlled experiments (see Maggioni et al., 2020). Engineers, chemists, public health specialists and smellscape researchers should engage in cross-disciplinary conversations and collaborations to establish clear objectives and methodologies for these databases. Involving artists and chemists/perfumers in the process can also contributed to the (re)creation of scents in the built environment, particularly in the heritage and museum contexts (see Verbeek, 2016; Spence, 2020b), where creating an experiential learning environment is essential.

Secondly, addressing social justice in odour control and management in urban planning and design would contribute to reducing odour pollution affecting minority communities, enhancing inclusivity for people who have partially or fully lost the sense of smell and fostering participation in decision-making processes concerned with removing or introducing smells to infrastructures and public spaces. Furthermore, promoting a real-time monitoring system and odour reporting platform—such as the OdourCollect app—would nurture a bottom-up, multi-level governance model. Creative methodologies drawn from artistic approaches could also facilitate participatory processes.

Lastly, research into advanced materials that can store and release natural smells and filter pollutants (i.e., VOCs and SVOCs) would advance the integration of smellscapes into current architectural and urban design practices. For example, recent research on nano materials by Lee and Jeon (2020) has shown the potential of polyacrylonitrile nanofiber membranes to filter gases and citrate smoke from burning cigarette paper to purify the air, thus contributing to safeguarding health and well-being in the built environment. These new materials could be used for the canopies in and around transport hubs, commercial kitchens and bus stops.

In addition to the above, the Covid pandemic has drawn attention to anosmia and parosmia. Initial studies suggest that up to 57% of Covid-19 patients experience some olfactory dysfunction (Agyeman et al., 2020; Moein et al., 2020). Subsequent to anosmia, an altered sense of smell, parosmia, occurs in over 50% of post-viral smell loss patients (Reden et al., 2006). This may result in either an inability or reticence to search for and sniff odours or the opposite (see Mahdawi, 2020; Rimmer, 2020). Köster et al. (2002) point out that a conscious coping mechanism for odours and the environment can potentially contribute to odour memory as a way to reduce the uneasiness experienced by people who experience anosmia. Odour education and the training of olfactorisation skills (ways to gain olfactory experiences via sensory cues beyond smelling) might increase well-being in the context of ongoing smell loss. Further research will be needed on designing an inclusive environment for people who experience a loss or changed sense of smell and on establishing environmental cues for olfactorisation.

## Author Contributions

JX, FA, and AR: contributed to conception and design of this perspective article. FA: introduction. JX and KM: methodological approaches in smellscape research. LS and CV: rediscovering designed smellscapes. AR: odour policy-making and the urban environment. JX, AR, and KM: outlook and concluding remarks. All authors wrote sections of the manuscript.

## Conflict of Interest

The authors declare that the research was conducted in the absence of any commercial or financial relationships that could be construed as a potential conflict of interest.
